# Reply to Dian et al

**DOI:** 10.1093/cid/ciy447

**Published:** 2018-05-31

**Authors:** Le Thi Phuong Thao, Ronald Geskus, Guy Thwaites

**Affiliations:** 1 Oxford University Clinical Research Unit, Ho Chi Minh City, Vietnam; 2 Centre for Tropical Medicine and Global Health, University of Oxford, United Kingdom

To the Editor—We thank Dr Dian and co-authors for their interest in our recent publication [1]. One of the limitations of clinical diagnostic and prognostic algorithms is that their performance may differ outside of the populations in which they were generated. Validation in different settings is required, and we are grateful to the authors for the application of their data from a cohort of Indonesian patients with tuberculous meningitis (TBM) to our prognostic tool, especially as it would have required sequential input of individual patient data from their cohort (n = 524).

Our prognostic tool did not perform well on the Indonesian cohort, although differences in the tool’s performance between the 2 cohorts are not altogether surprising. As indicated by the authors, patients in the Indonesian cohort had more severe disease at the start of treatment than the Vietnam cohort; but baseline Medical Research Council disease severity grade were included in our prognostic models and thus would be unlikely to explain the tool’s poor performance in the Indonesian cohort.

Instead, recent publications allow comparison of measures and determinants of cerebrospinal fluid (CSF) inflammation and outcomes between the 2 populations and suggest there may be biological differences between them that may lead to different prognostic variables [2, 3]. For example, leukotriene A4 hydrolase genotype influenced CSF inflammation and survival in the Vietnamese cohort but not the Indonesian cohort. In addition, we have previously shown high CSF neutrophil numbers are associated with the culture of *Mycobacterium tuberculosis* from CSF in Vietnam [4], but unlike the Indonesian cohort, lower neutrophil numbers were associated with death [5]. Studies of TBM in human immunodeficiency virus coinfected patients in South Africa have also reported that high CSF neutrophil numbers predicted positive CSF mycobacterial cultures and, intriguingly, the development of central nervous system immune reconstitution inflammatory syndrome [6, 7]. These studies highlight shared and discrepant pathophysiological mechanisms between populations with TBM, which may be driven by genomic variation in the bacteria or their hosts and may undermine attempts to define universally applicable clinical prognostic models.

The authors suggest that CSF culture and neutrophil counts might be more reliable predictors of death than those we modeled. CSF cultures were not included in our models because the results are not available before the start of treatment and therefore are not of clinical utility when defining prognosis at diagnosis. CSF neutrophil count is strongly correlated with CSF lymphocyte count and, for this reason, was not included in the model; although as described above, neutrophils do appear important in TBM pathophysiology. However, we thank the authors for exposing a fault in the web-based tool: CSF lymphocyte counts >500/µL cannot currently be entered. We have therefore modified the tool to allow submission of higher counts.

## Note

***Potential conflicts of interest.*** All authors: No reported conflicts of interest. All authors have submitted the ICMJE Form for Disclosure of Potential Conflicts of Interest. Conflicts that the editors consider relevant to the content of the manuscript have been disclosed.
